# Isolation and Identification of Multidrug-Resistant *Klebsiella pneumoniae* Clones from the Hospital Environment

**DOI:** 10.3390/pathogens12050634

**Published:** 2023-04-23

**Authors:** María Guadalupe Córdova-Espinoza, Silvia Giono-Cerezo, Erika Gabriela Sierra-Atanacio, Alejandro Escamilla-Gutiérrez, Eduardo Carrillo-Tapia, Laura Isabel Carrillo-Vázquez, Felipe Mendoza-Pérez, Martha Leyte-Lugo, Raquel González-Vázquez, Lino Mayorga-Reyes, Rosa González-Vázquez

**Affiliations:** 1 Escuela Nacional de Ciencias Biológicas, Instituto Politécnico Nacional, Departamento de Microbiología, Prolongación de Carpio y Plan de Ayala S/N, Col. Casco de Santo Tomas, Alcaldía Miguel Hidalgo, Mexico City 11340, Mexico; 2 Escuela Militar de Graduados de Sanidad SEDENA, Laboratorio de Inmunologia, Batalla de Celaya 202, Col. Lomas de Sotelo, Alcaldía Miguel Hidalgo, Mexico City 11200, Mexico; 3 Instituto Mexicano del Seguro Social, Hospital General “Dr. Gaudencio González Garza”, Centro Medico Nacional La Raza, Privada de las Jacarandas, S/N, Col. La Raza, Alcaldía Azcapotzalco, Mexico City 02990, Mexico; 4 Colegio de Ciencias y Humanidades, Universidad Autónoma de la Ciudad de México, Avenida de la Corona 320, Col. Loma de la Palma, Alcaldia Gustavo a Madero, Mexico City 07160, Mexico; 5 Posgrado en Ciencia Genómicas, Universidad Autónoma de la Ciudad de México, San Lorenzo 290, Col. Del Valle, Alcaldía Benito Juárez, Mexico City 03130, Mexico; 6 Laboratorio de Biotecnología, Departamento de Sistemas Biológicos, Universidad Autónoma Metropolitana Unidad Xochimilco, Calzada del Hueso 1100, Col. Villa Quietud, Alcaldía Coyoacán, Mexico City 04960, Mexicolmayorga@correo.xoc.uam.mx (L.M.-R.); 7 Laboratorio de Biotecnología, Departamento de Sistemas Biológicos, CONACYT-Universidad Autónoma Metropolitana Unidad Xochimilco, Calzada del Hueso 1100, Col. Villa Quietud, Alcaldía Coyoacán, Mexico City 04960, Mexico; 8 Instituto Mexicano del Seguro Social, Unidad Médica de Alta Especialidad, Hospital de Especialidades “Dr. Antonio Fraga Mouret”, Centro Medico Nacional La Raza. Seris y Zaachila S/N, Col. La Raza, Alcaldía Azcapotzalco, Mexico City 02990, Mexico

**Keywords:** MDR, XDR, PDR, *Klebsiella pneumoniae*, MLST, opportunist, healthcare-associated infection, outbreak

## Abstract

Global dispersion, hospital outbreaks, and lineage relationships between emerging antibiotic-resistant strains such as *Klebsiella pneumoniae* are of public health interest. This study aimed to isolate and identify *K. pneumoniae* clones from third-level healthcare hospitals in Mexico to establish their multidrug-resistant phenotype, phylogeny, and prevalence. Biological and abiotic surface samples were used to isolate *K. pneumoniae* strains and to test their antibiotic susceptibility to classify them. The housekeeping genes: *gapA*, *InfB*, *mdh*, *pgi*, *phoE*, *ropB*, and *tonB* were used for multilocus sequence typing (MLST). Phylogenetic networks were constructed with 48 strains. Isolated strains (93) were mainly from urine and blood, 96% were resistant to ampicillin as expected, 60% were extended-spectrum β-lactamases (ESBL), 98% were susceptible to ertapenem and meropenem and 99% were susceptible to imipenem, 46% were multi-drug resistant (MDR), 17% were extensively-drug resistant (XDR), 1% were pan-drug resistant (PDR), and 36% were not classified. The *tonB*, *mdh*, and *phoE* genes were the most variable, and the *InfB* gene showed positive selection. The most prevalent sequence types (STs) were ST551 (six clones), ST405 (six clones), ST1088 (four clones), ST25 (four clones), ST392 (three clones), and ST36 (two clones). ST706 was PDR, and ST1088 clones were MDR; neither of these STs has been reported in Mexico. The strains analyzed were from different hospitals and locations; thus, it is important to maintain antibiotic surveillance and avoid clone dissemination to prevent outbreaks, adaptation to antibiotics, and the transmission of antibiotic resistance.

## 1. Introduction

*K. pneumoniae* is an opportunist, emerging microorganism from the ESKAPE group formed by the *Enterococcus faecium*, *Staphylococcus aureus*, *K. pneumoniae*, *Acinetobacter baumannii*, *Pseudomonas aeruginosa*, and *Enterobacter* species, which are responsible for nosocomial infections [1]. It is also responsible for healthcare-associated infections (HAI) such as central line-associated bloodstream, surgical site, catheter-associated urinary tract infections, and ventilator-associated pneumonia. *K. pneumoniae* is frequently isolated from immunosuppressed patients in the intensive care unit (ICU). The most important virulence factors, which allow it to evade the immune response and promote the microorganism’s establishment, are adhesins (fimbriae), capsular polysaccharides (serotypes K1 and K2), siderophores, and lipopolysaccharide [2,3].

Worldwide, bacterial resistance is an increasingly severe problem, as well as ESBL dissemination [4] and different subgroups of CTX-M (a group of enzymes of class A ESBL), which generates cephalosporin resistance, and transferable resistance mechanisms to quinolones and aminoglycosides. Carbapenem resistance transference by plasmids is important because they can be transmitted to other bacteria, such as Enterobacteriaceae and other non-fermentative bacilli. The recent isolation of *K. pneumoniae* carbapenemase KPC-producing bacteria (resistant to all β-lactams, cephalosporins, penicillin, and monobactams) at ICUs worldwide was associated with resistance to other antimicrobial agents, which were contained in the same plasmid [5]. Therefore, it is essential to investigate global dispersion, hospital outbreaks, and lineage relationships between resistant clones from hospital and community environments and compare endemic and pandemic clones through molecular typing methods such as MLST.

The genetic diversity of *K. pneumoniae* has been studied previously, and the most dominant ST was from China [6]. Other reports show that nosocomial clones such as ST258 acquire resistance to plasmids easily [7]. ST405 is a recently reported clone of fast dissemination and is classified as high-risk [8]. This study aimed to isolate and identify *K. pneumoniae* clones from third-level healthcare hospitals in Mexico to establish their multidrug-resistant phenotype, phylogeny, and prevalence. Our results showed that there are clones present in Mexico that were previously reported in faraway countries, and there are other clones that have not yet been reported. One PDR clone was identified. The strains analyzed were from different hospitals and locations, which indicates that it is crucial to maintain antibiotic surveillance and avoid clone dissemination to prevent outbreaks, adaptation to antibiotics and the transmission of antibiotic resistance.

## 2. Materials and Methods

### 2.1. Isolation and Identification of K. pneumoniae Clones

*K. pneumoniae* clones from urine, blood, respiratory secretions, vaginal fluids, wounds, catheter tips, and abiotic surface samples were isolated from different third-level healthcare hospital units and one family clinic from Mexico City (classified as hospital I, II, III, and IV). Hospital I and II are found in Azcapotzalco, hospital III in Iztapalapa, and hospital IV in Tlalpan. The isolation was performed in their respective hospital clinical laboratories. This study did not involve humans; it was an in vitro study using isolated strains.

In the hospitals, isolates were identified by colony, microscopic morphology, and conventional biochemical tests, including indole, lysine, ornithine, lactose fermentation, Voges-Proskauer/methyl Red, gas production, Simmons citrate, urea, o-nitrophenyl-β-D-glucopyranoside, KCN, catalase, oxidase, and motility [9,10]. The cryopreservation of strains was carried out in BHI broth with 20% (*v/v*) glycerol and stored at −20 °C. Strains in this study were deposited in the medical bacteriology culture collection of Escuela Nacional de Ciencias Biologicas at Instituto Politecnico Nacional, Mexico City.

### 2.2. K. pneumoniae Strains Antibiotic Resistance Test

Antibiotic resistance of the isolated *K. pneumoniae* strains was determined by the Vitek2^®^ system (Biomérieux, Lyon, France) based on the minimum inhibitory concentration (MIC) [11]. The tested antibiotics were ampicillin (AMP), cefazolin (CFZ), trimethoprim/sulfamethoxazole (SXT), ampicillin/sulbactam (SAM), cefepime (FEP), ceftriaxone (CRO), tobramycin (TOB), gentamicin (GEN), nitrofurantoin (NIT), ciprofloxacin (CIP), piperacillin/tazobactam (TZP), amikacin (AMK), meropenem (MEM) ertapenem (ETP), and imipenem (IMP) [11]. MDR strains were defined as those strains which were resistant to the therapeutic election categories [11]. XDR strains were classified as those strains which were resistant to at least one agent in all or at least two therapeutic election categories [11]. PDR strains were defined as those strains resistant to all agents in all antimicrobial categories [11]. Clinical isolates with resistance to one antibiotic in less than three categories were considered unclassifiable. Control strains were *K. quasipneumoniae* ATCC 700603 [12] and *E. coli* ATCC 25922.

### 2.3. Confirmatory Extended-Spectrum β-lactamases (ESBL)

An ESBL confirmatory test was performed using the Double Disc Synergy Test employing ceftazidime (CAZ) (30 μg), CAZ-clavulanate (30 μg/10 μg), cefotaxime (CTX) (30 μg), and CTX-clavulanate (30 μg/10 μg) disks. Positive results were considered when an increase in the diameter of the inhibition halo (≥5 mm) was observed around the CTX or CAZ-clavulanate, compared to one without clavulanate [11]. Quality control was performed using *K. quasipneumoniae* ATCC 700603 and *E. coli* ATCC 25922.

### 2.4. Molecular Characterization

To perform the MLST scheme, 48 isolated and selected strains were compared against 2317 STs reported in the MLST database (http://bigsdb.pasteur.fr/, accessed on 6 May 2022). The criteria used to select the 48 isolates were: hospital, origin of the sample (mainly urine and/or blood culture), and the presence or absence of ESBL. The gDNA of selected clones was extracted by using the guanidine thiocyanate method. The MLST scheme (including *gapA*, *InfB*, *mdh*, *pgi*, *phoE*, *ropB*, and *tonB* genes) has been described previously by Diancour [13]. PCR products were purified using PureLink Quick gel extraction (Invitrogen, Carlsbad, CA, USA) and an EZ-10 Spin Column PCR purification Kit (BioBasic, Markham, ON, Canada). Sequencing PCR protocol was carried out using ABI PRISM Big dye Terminator v3.1 Cycle Sequencing Kits (Applied Biosystems, Foster City, CA, USA). ABIPRISM 3730XL equipment was used to read sequencing reactions.

The eBURST v3 program was used to obtain CC [14]. Sequence type analysis was performed using the eBURST v3, START2 v 0.9.0, and DNAsp v5.10 programs [15,16]. The ExPASY (www.expasy.org, accessed on 15 June 2022) website was also used for amino acid translation.

The network plot was performed in a Python v3.6.7 environment through the platform Jupyter Notebook v6.0.3 by an in-house python script using the Matplotlib v3.0.3, Pandas v1.0.4, Pandas v1.0.4 and Numpy v1.18.5 packages. We applied edge bundling on maps, which uses a self-organizing approach to bundling [17]. The nodes and edges were obtained using the program Phyloviz v2.0, which uses the goeBURST algorithm, which is a refinement of the eBURST algorithm by Feil et al. [14].

### 2.5. Phylogenetic Analysis

The multiple sequence alignments (MSAs) and phylogenetic tree of the multilocus sequence including the *gapA, InfB, mdh, pgi, phoE, ropB*, and *tonB* genes, were generated using the Next Generation Phylogeny.fr web service available at https://NGPhylogeny.fr [18] (accessed on 24 March 2023). We used the “One-Click Workflow” tool from NGPhylogeny.fr, which offers a customizable platform for phylogenetic analysis and MAFFT (Multiple Alignment using Fast Fourier Transform) [19]. The MAFFT alignment was curated using BMGE (Block Mapping and Gathering with Entropy) [20] and FastME (Fast Minimum Evolution) [21] was used to produce the tree file. FastME uses distance algorithms to infer phylogenies. The newick file was processed in a Python v3.6.7 environment through the platform Jupyter Notebook v6.0.3 and the final tree was generated by an in-house python script using the Matplotlib v3.0.3, Pandas v1.0.4 and Biopython v1.79 packages. In addition, information associated (hospital, resistance, ESBL production, clinical sample, hospital service) with the strains illustrated in the phylogenetic tree was added.

## 3. Results

### 3.1. K. pneumoniae Strains Identification and Susceptibility Test

**Figure 1:**
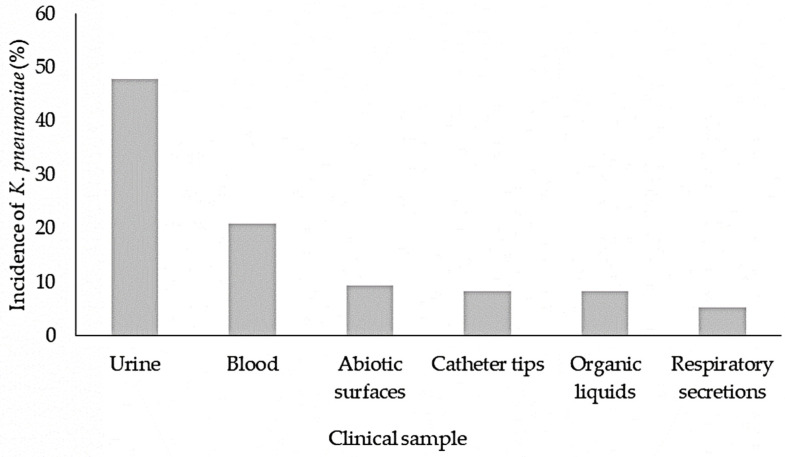
Incidence of *K. pneumoniae* in clinical samples.

Ninety-three *K. pneumoniae* strains were isolated from different samples (Figure 1). Specifically, 70% were isolated from hospital I, 12% from hospital II, 5% from hospital III, and 13% from hospital IV. All of them were Gram negative bacteria, catalase +, lysine +, gas production +, lactose fermentation +, Voges-Proskauer +, Simmons citrate +, urea +, o-nitrophenyl − β D-galactopyranoside (ONPG) +, KCN +, oxidase −, indole −, ornithine −, methyl red −, and motility −.

**Figure 2:**
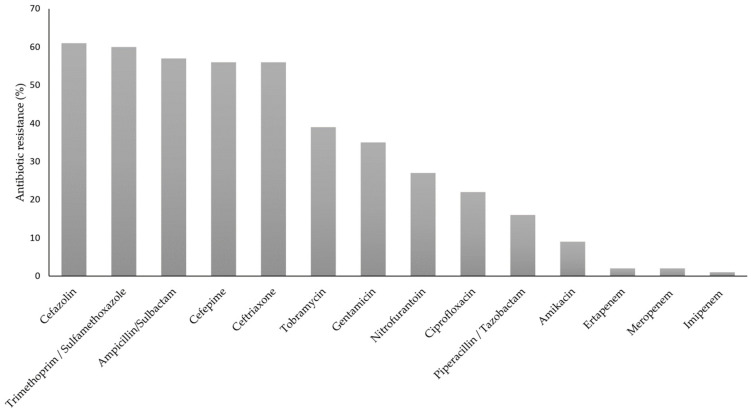
Antibiotic resistance of isolates of the *K. pneumoniae* isolated strains.

The antibiotic resistance of the 93 *K. pneumoniae* strains is shown in Figure 2, in which it can be observed that the higher percentage of resistance belongs to AMP. On the other hand, 60% of the population (56/93) was classified as ESBL-producing strains.

The isolated strains were classified as follows: 46% (43/93) were MDR, twenty-four from Hospital I, ten from Hospital II, one from Hospital III, and eight from hospital IV; 17% (16/93) were XDR, ten from Hospital I, one from Hospital II, and two and three from Hospital III, and IV, respectively. PDR strain (1%, 1/93) was isolated from Hospital IV. A total of 36% of the population (33/93) was not classified.

### 3.2. MLST Results

The genetic diversity of the 48 *K. pneumoniae* isolates, the control strain, and the allelic profile of the seven housekeeping genes from each clinical isolate and their STs are shown in Table 1, where *tonB*, *mdh*, and *phoE* genes showed the most variation.

**Figure 3:**
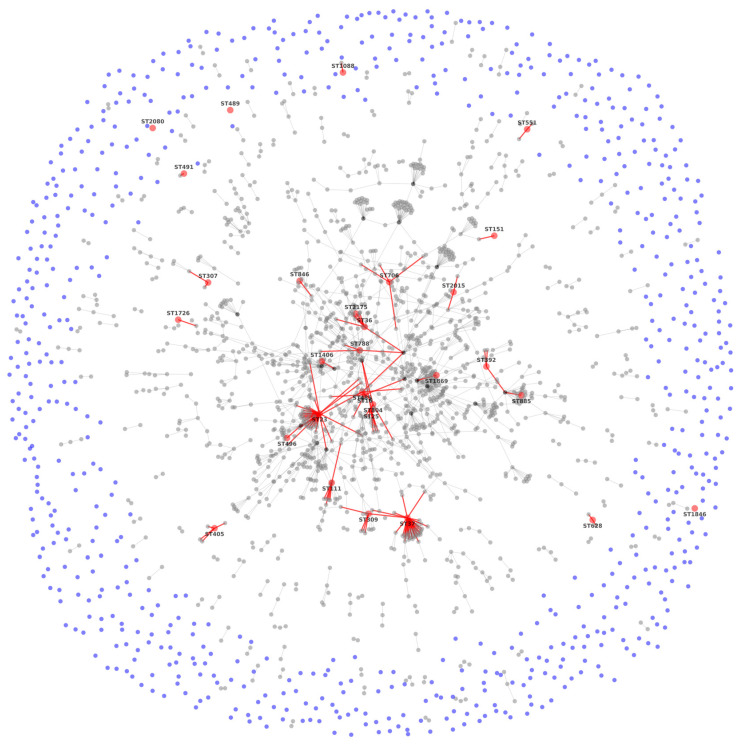
Snapshot population of the isolated *K. pneumoniae*. eBURST comparative analysis. Nodes without interaction are shown as blue dots; nodes with a value of interaction greater than 10 are shown in black. The STs identified in this study together with their interactions with the closest nodes are shown in red.

In this study, 29 STs were identified among the clinical strains isolated, (Figure 3). The *K. pneumoniae* ATCC 700603 belonged to ST489 S [22,23]. The Burst algorithm identified groups of CC and allowed us to recognize the founding genotype of each group. Additionally, a population snapshot analysis was obtained and showed a clonal structure (IA = 0.572 (*n* = 48) (START2 V.1.0.5)).

CC denomination was assigned according to each group’s founding ST, and it is considered that a CC is composed of isolations that have six/seven identical loci. There was a total of 183 CC. The principal clonal complex (CC11) is observed in the center of the snapshots, and this complex showed variation in different positions as follows: 47 SLV (single-locus variant), 43 DLV (double-locus variant), 123 (triple-locus variants) TLV and 688 SLV, where 18 ST were found. The CC to which the isolated strains belong are CC147 (ST885, ST392); CC628 (ST628); CC1088 (ST1088); CC551 (ST551); CC2054 (ST10726); CC491 (ST491); CC405 (ST405), and CC307 (ST307). The individual STs (singletons) that were not part of a CC were ST2080, ST1846, and ST489.

Twenty-three STs (79%) were from one strain, and six STs (21%) were from two or six isolates. Predefined STs were ST551, and ST405 (12.5%, 6/48), then ST25 and ST1088 (8.3%), ST392, which was present straight away (6.3%, 3/48%), and finally, ST36 (4.1%, 2/48).

### 3.3. Sequence Analysis

The characteristics and polymorphism of each gene are shown in Table 2. Each gene’s number of alleles varied from four (*gapA* and *ropB*) to seventeen (*tonB*), and the number of polymorphic sites matched with each gene mutation. Values of nucleotide diversity per site (π), from 0.00463 (*pgi*) to 0.02794 (*gapA*) and average nucleotide differences per site (θ) from 0.00364 (*gapA*) to 0.01331 (*rpoB*) were <1. G + C content was over 50% in the seven genes, with a range from 55.77% (*mdh*) to 64.66% (*tonB*).

Polymorphic changes match with the mutations; most housekeeping genes did not show mutations on the different triplet positions, with the number of non-synonymic (dN) and synonymic (dS) substitution of amino acid per site. The dN/dS relation for most of the genes was <1, indicating that there was no strong selective pressure over the genes, except for the *InfB* gene (Table 2).

### 3.4. Phylogenetic Analysis

**Figure 4:**
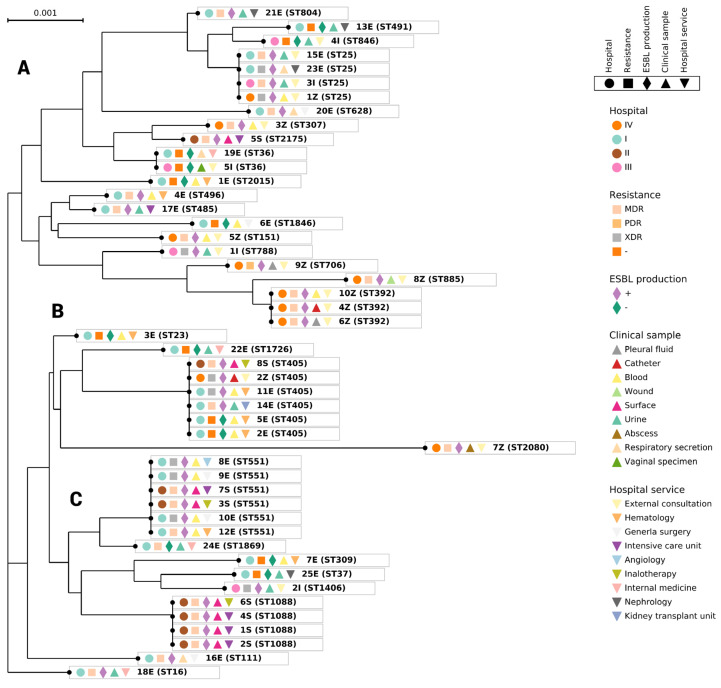
Phylogeny of the *K. pneumoniae* isolated strains. The length of the branches of the phylogenetic tree indicates the evolutionary change of each isolate. (**A**–**C**) represent the three clades found.

Phylogenetic analysis was performed with 48 isolated strains. Branch lengths indicates the evolutive changes (Figure 4). The MDR and XDR of the most prevalent strains were: ST551 (3/6) and (3/3); ST405 (2/6) and (2/6); ST25 (2/4) and (2/4); ST1088 (4/4) and (0), ST392 (3/3) and (0); ST36 (0) and (0) (Figure 4).

The phylogenetic tree shows three clades; the A clade includes 50% (24/48), the B clade includes 40% (19/48), and the C clade includes 10% (5/48) of the isolated strains. In clade A, 50% of the strains belonged to the most prevalent STs (ST392, ST551, and ST36). In general, most of the strains in the A clade were isolated from external consultation. The other most prevalent STs (405, 1088 and 25) were found in the B clade. ST 551 and 36 were found at the same phylogenetic distance (Figure 4), and the other most prevalent STs showed different phylogenetic distances (Figure 4). There was no relation between antibiotic resistances within the phylogenetic group’s formation.

The ST706 clone was PDR, and ST1088 was MDR. These strains have yet to be reported in Mexico, but ST706 was previously reported in Germany in 1997, where it was isolated from the clinical urinary tract [24].

## 4. Discussion

According to the latest data from the Centers for Disease Control and Prevention of the United States, ESKAPE group bacteria are responsible for two-thirds of all HAI and play a significant role in worldwide mortality. *K. pneumoniae* belongs to this group. In Mexico, *K. pneumoniae* is classified as one of the three main HAI aetiological agents and the second most reported microorganism in outbreaks [25]. In fact, the 2016 RHOVE (hospital network of epidemiological surveillance in Mexico) reported that *K. pneumoniae* held second place in isolation frequency in bloodstream infections and fourth place in urinary system infections [26,27]. The 2022 RHOVE report, emphasizes that *K. pneumoniae* is the third leading cause of HAI [25,28].

In this study, 93 clinical strains confirmed as *K. pneumoniae* were isolated from four hospital centers located in different geographical regions of Mexico City. Most of the samples obtained from different services of hospital I were from blood and urine cultures. In the case of hospital II, most of the samples were from abiotic surfaces. Hospital III showed a high frequency of isolation from urine cultures; however, these samples were mainly from external consultation, which implies that the infection was acquired in the community, and it does not present importance in terms of HAI. However, it is indicative of the dissemination of the strains in the environment, possibly determined by human activity. Hospital IV had a higher frequency of isolation from blood culture samples, suggesting a complexity in the treatment of infections by *K. pneumoniae*, because strains in the bloodstream can spread to any organ and cause sepsis.

It has been reported that the isolation of *K. pneumoniae* strains have wide variation by geographic region in the specimen source most frequently associated with ESBL non-carbapenem-resistant (non-CRE) phenotypes. Karlowsky et al. (2022) [29] have reported that in most regions of Asia, Europe, Australia, New Zealand, Latin America including Mexico, the Middle East, Africa, the United States of America, and Canada, ESBL non-CRE phenotype isolates were found more frequently in samples from ICU patients than non-ICU patients and from patients with a hospital length of stay at time of specimen collection ≥ 48 h. They also reported that the presence of ESBL non-CRE phenotypes increased significantly in Latin America, USA and Canada in 2018 and 2019. On the other hand, *K. pneumoniae* strains from carbapenem-resistant (CRE) clinical isolates have been reported in Mexico. In fact, they have been classified as KPC-2 (ST258) [30], KPC-3 (ST258) [31] and NDM-1 (ST5) producing strains [30,32]. In addition, an elevated incidence and prevalence of CRE-fecal carriage has been reported in Mexican hospitals, which highlights the need to establish and improve control programs to avoid nosocomial spread [33].

The increase in resistance in *K. pneumoniae* has risen due to the indiscriminate use of antibiotics in the treatment of infections caused by this bacterium, and this is becoming a worldwide problem. Antibiotic resistance is more frequently associated with the expression of genes present in plasmids, transposons, and integrons, which are key elements in the horizontal transmission of genetic information, because they can spread antibiotic resistance between members of the same or different species [34].

The antibiotic resistance reported in this study was towards different antibiotic groups such as β-lactams, cephalosporins, aminoglycosides, and sulfas. On the other hand, most isolated strains were susceptible to ertapenem and meropenem (98%), and imipenem (99%), as has been reported for strains isolated in Latin America and Mexico [35]. The susceptibility to carbapenems can be used as a strategy in hospitals to guide the clinician to use all antibiotics differently from carbapenems to which the strain is susceptible, instead of using carbapenems as the first treatment due to high susceptibility. The use of this strategy will make it possible to modify the guidelines of empirical therapy so as not to generate selection pressure [36] in non-CRE strains and thus avoid resistance to carbapenems.

Ostria-Hernandez et al. (2018) [35] have reported *K. pneumoniae* strains resistant to cephalosporins, including CRO (97.8%), FEP (58.8%), and TZP (44.7%) are present in various Latin American countries. In this study, we found a low percentage of resistance regarding CRO and TZP, which was like FEP.

In Mexico, MDR strains (83%) have been reported, but no PDR strains have been reported [35]. In the present study, the frequency of MDR strains was 46%, whereas one PDR strain was found (ST706) as well as some XDR. Infections caused by XDR and PDR in *K. pneumoniae* represent an emerging threat due to the high mortality rate associated with them [37].

The association index confirmed that the studied population was a clonal type; in other words, the isolated strains are genetically related. Diverse clones had been previously reported in countries far away from Mexico [35,38], allowing us to visualize the rapid dissemination of some clones of *K. pneumoniae* among hospitals no matter the location or where they were isolated (in biological samples and on hospital surfaces).

In clade A of the phylogenetic tree, the predominant ST was ST551. The strain was isolated from two different hospitals from patients and surface samples. This strain was held responsible for an outbreak in Azcapotzalco, Mexico City. This clone was also isolated in 2011–2013 in Japan, according to Higashino et al. [39], who reported that *K. pneumoniae* was the most frequent bacteria isolated from urine and respiratory secretion samples. *K. pneumoniae* ST392 was also reported by Bocanegra-Ibarias in 2017 [13,40] in Mexico, and it was classified as endemic in the hospital because it was detected during an 11-month surveillance. ST309 and ST307 were also obtained; these strains were isolated in Mexico (2014–2015) and South Africa (2014–2016), respectively [40,41]. All the clones were MDR; however, these clones have been reported as harboring *blaKPC*, *blaOXA*-48, and *blaNDM-1* genes. In contrast, the *K. pneumoniae* ST309 detected has not been related to the carbapenemases gene; *K. pneumoniae* ST706 was the only PDR strain and was isolated from hospital IV. This strain has not been reported since 1997 in Germany [24]. Thus, it will be important to follow its outbreaks, analyze the whole genome of this strain, and keep it under surveillance to avoid dissemination.

In the B clade, six clones of ST405 were predominant. They were isolated from samples of urine and blood obtained from hospitals I, II, and IV in the period from 2011 to 2015. The ST405 clones were MDR and XDR. The ST405 clone was responsible for outbreaks in Spain in 2010–2012 [42] and in Italy, where it caused an outbreak in 2013–2014 [43]. Although the strains had different virulence factors, this could be because the samples obtained were taken from community waters near the hospitals. These strains are susceptible to antibiotics; hospital strains are resistant, but wild strains express more virulence factors [44]. Another predominant ST of the B clade was ST25 (four clones), isolated from hospitals I, III, and IV, in 2013–2015. ST25 was reported in Japan in 2008 and in China in 2014–2015 [39]. ST1088 was found only on abiotic surfaces in hospital II in 2013, suggesting that adequate surveillance and preventive measures at the hospitals are needed; for example, rotating the disinfecting solutions occasionally to prevent dissemination to patients in the UCI. Because ST1088 is MDR and EBLS positive, it is recommended to maintain surveillance as these strains can quickly obtain genetic material for carbapenem resistance.

It is important to highlight that all the strains isolated from abiotic surfaces (four strains of ST1088, two strains of ST 551, one strain of ST2175 and one strain of ST405), were from hospital II. In the case of ST1088 and ST2175, three and one strains, respectively, were isolated from abiotic surfaces of the intensive care unit, where the use of antibiotics is usually much higher compared to medical and surgical wards [45] and where the isolation of ESKAPE pathogens in high levels has been reported [46].

STs 551 and 405 were also found in biological samples mainly isolated from blood and one sample from urine, all of which came from hospital I. Hospitals I and II belong to the same hospital area, hence contamination from infected patients or the environment could be likely, in which sewage system and other factors could be involved [47], highlighting the need to establish policies aimed at reducing acquired resistance to antibiotics [47].

Additionally, one strain, ST405, and another, ST551, were isolated from an inhalotherapy service at hospital II, which may indicate contamination from patients to hospital abiotic surfaces. As has been suggested previously, in hospital services: (1) bacterial pathogens circulating in hospitals, especially the ESKAPE group pathogens, and (2) the microbiome of various abiotic surfaces in hospitals indicates the need for epidemiological monitoring to establish an adequate system of cleaning and disinfection of possible abiotic surfaces at risk of contamination to avoid the spread of antibiotic resistant strains.

## 5. Conclusions

Our results showed that there are clones present in Mexico that were previously reported in faraway countries; however, ST706 and ST1088 clones have yet to be reported in Mexico City, and in the case of ST1088, it has yet to be reported in another country. The ST706 clone was PDR. The strains analyzed were from different hospitals and locations. Thus, it is important to maintain antibiotic surveillance and clone dissemination to prevent spread through patients, abiotic surfaces and between hospitals to reduce outbreaks and the possible transfer of resistance to antibiotics between microorganisms such as those of the ESKAPE group, which are responsible for severe and deadly infections with increasing morbidity and mortality worldwide and which are greatly threatening public health.

## Figures and Tables

**Table 1:** Allelic profile of housekeeping genes from *K. pneumoniae* clones isolated from different hospital samples.

				Allelic Profile				
Strain	*gapA*	*rpoB*	*mdh*	*pgi*	*phoE*	*InfB*	*tonB*	ST
*K. quasipneumoniae* ATCC 700603	18	52	26	71	98	22	51	489
1E	2	1	1	3	8	1	15	2015
2E	2	1	62	3	10	4	110	405
3E	2	1	1	1	9	4	12	23
4E	2	1	1	6	7	4	12	496
5E	2	1	62	3	10	4	110	405
6E	2	1	164	1	7	4	303	1846
7E	2	9	2	1	13	1	10	309
8E	3	1	1	1	9	4	135	551
9E	3	1	1	1	9	4	135	551
10E	3	1	1	1	9	4	135	551
11E	2	1	62	3	10	4	110	405
12E	3	1	1	1	9	4	135	551
13E	51	1	5	1	9	4	13	491
14E	2	1	62	3	10	4	110	405
15E	2	1	1	1	10	4	13	25
16E	2	1	5	1	17	4	42	111
17E	2	1	1	1	7	1	12	485
18E	2	1	2	1	4	4	4	16
19E	2	1	2	1	7	1	7	36
20E	2	60	11	1	4	8	24	628
21E	2	1	2	1	1	4	13	804
22E	2	1	1	117	10	4	18	1726
23E	2	1	1	1	10	4	13	25
24E	3	3	1	1	9	1	4	1869
25E	2	9	2	1	13	1	16	37
1S	2	1	1	10	1	1	76	1088
2S	2	1	1	10	1	1	76	1088
3S	3	1	1	1	9	4	135	551
4S	2	1	1	10	1	1	76	1088
5S	2	1	2	1	213	1	7	2175
6S	2	1	1	10	1	1	76	1088
7S	3	1	1	1	9	4	135	551
8S	2	1	162	3	10	4	110	405
1I	2	4	2	1	7	1	12	788
2I	3	3	1	1	13	1	79	1406
3I	2	1	1	1	10	4	13	25
4I	2	1	97	1	9	4	13	846
5I	2	1	2	1	7	1	7	36
1Z	2	1	1	1	10	4	13	25
2Z	2	1	62	3	10	4	110	405
3Z	4	1	2	52	1	1	7	307
4Z	3	4	6	1	7	4	40	392
5Z	4	1	32	1	7	4	10	151
6Z	3	4	6	1	7	4	40	392
7Z	4	5	88	1	1	94	23	2080
8Z	3	4	6	1	7	4	13	885
9Z	2	4	1	1	7	4	4	706
10Z	3	4	6	1	7	4	40	392

**Table 2:** Characteristics and polymorphism of the housekeeping genes from the clinical isolates of *K. pneumoniae*.

Gene	PCR (bp)	Haplotype	Polymorphic Sites	Total Mutations	π	θ	G + C	dN	dS	dN/dS
*gapA*	450	4	3	3	0.02794	0.00364	0.5615	0.0000	0.0132	0.0000
*InfB*	318	6	7	7	0.00797	0.00964	0.6140	0.0097	0.0037	2.6548
*mdh*	477	10	21	21	0.00526	0.00684	0.5577	0.0086	0.0145	0.5944
*pgi*	432	6	6	6	0.00463	0.00608	0.5733	0.0042	0.0056	0.7531
*phoE*	420	9	9	9	0.00728	0.00788	0.5585	0.0000	0.0311	0.0000
*rpoB*	501	4	13	13	0.01331	0.01331	0.5415	0.0128	0.0147	0.8713
*tonB*	414	17	16	16	0.01027	0.01143	0.6466	0.0050	0.0257	0.1961

## Data Availability

Not applicable.
